# Faunistic Composition and Spatial Distribution of Scorpions in North Khorasan Province Northeast of Iran

**Published:** 2019-12-31

**Authors:** Faranak Firoozfar, Abedin Saghafipour, Hassan Vatandoost, Mulood Mohammadi Bavani, Masoumeh Taherpour, Nahid Jesri, Mahmood Yazdani, Kourosh Arzamani

**Affiliations:** 1 Vector-borne Diseases Research Center, North Khorasan University of Medical Sciences, Bojnurd, Iran; 2 Department of Public Health, Faculty of North Khorasan University of Medical Sciences, Bojnurd, Iran; 3 Department of Public Health, Faculty of Health, Qom University of Medical Sciences, Qom, Iran; 4 Department of Medical Entomology and Vector Control, School of Public Health, Tehran University of Medical Sciences, Tehran, Iran; 5 Department of Medical Entomology and Vector Control, School of Public Health, Urmia University of Medical Sciences, Urmia, Iran; 6 Remote Sensing and GIS Center, Shahid Beheshti University, Tehran, Iran

**Keywords:** Scorpions, Spatial distribution, Geographic information system (GIS), Iran

## Abstract

**Background:**

Scorpions pose one of the most important public health and medical problems in tropical and subtropical regions of the world, especially in developing countries. This study was conducted to determine the fauna and spatial distribution of scorpions.

**Methods:**

In this descriptive study, scorpions were captured using ultra-violet (UV) light, pitfall traps and digging methods in North Khorasan Province, northeastern Iran in 2017. After being encoded, the collected scorpions were stored in plastic containers of 70% ethanol and then transferred to the medical entomology lab of Tehran University of Medical Sciences for species identification based on morphological keys. In addition, Arc Geographic Information System (GIS) 9.3 software was utilized for mapping spatial distribution of scorpions.

**Results:**

Overall, 143 scorpions were captured and identified. All of collected scorpions belonged only to Buthidae family. They were also classified into four genera (*Androctonus*, *Mesobuthus*, *Odontobuthus*, *Orthochirus*) and five species: *M. eupeus* (59.44%), *A. crassicauda* (16.78%), *O. doriae* (12.59), *M.* (*Olivierus*) *caucasicus* (9.09%), and *O. farzanpayi* (2.10%). Furthermore, spatial distribution of scorpions was performed in this area.

**Conclusion:**

Regarding the diversity, high frequency and wide geographical distribution of scorpions and their long-term seasonal activity in this area, the probability of occurrence of scorpion sting is high. Therefore, in order to prevent the occurrence of this public health problem, health educational programs be implemented by health- care providers in the area.

## Introduction

Scorpions (Arachnida: Scorpions) are one of the oldest terrestrial arthropods, dating back to about 450 million years ago (1). These animals are nocturnal, meaning that they leave their shelter at night for hunting and other biological activities but in daytime, they are hidden in their sanctuaries including seams and gaps of walls, underground holes, undersides of rocks, under the leaves and shells of trees, etc. Scorpions have venomous stings that they use to capture insects and defend themselves against natural enemies (2). Scorpions feed on insects, spiders, mites and some small vertebrates, while some species of them are cannibals (3, 4). These creatures often live in desert areas, but some of their species are found under rocks in mountainous and forest areas. Non-digger and semi-digger scorpions can enter human habitats (2).

Although the scorpions do not transmit any pathogens and parasitic agents to humans, they have always been one of human concerns due to their venomous and deadly stings, as well as scary appearance. Some species of scorpions are medically important and cause relatively high mortality in some areas of the world (5). Therefore, scorpionism turns out to be one of the most important public health and medical issues in tropical and subtropical regions of the world, especially in developing countries (6). In Iran, in addition to the anxiety and concern caused by scorpion sting, its high cost of treatment, and the resulting health-related consequences, deaths from scorpion stings are also reported annually (7). According to the Centers for Disease Control and Prevention (CDC) of Iranian Ministry of Health and Medical Education, approximately 40000–50000 cases of scorpionism occur annually in Iran (8, 9).

Systematically in the world, the scorpions are classified into six super families, 13 families, 18 subfamilies and 10 tribes and more than 2,000 species (10, 11). A review of the previously published articles in this regard showed that there are three families of scorpions in Iran, including Buthidae, Scorpionidae, Hemiscorpidae, identified in the form of 19 genera and 59 species (12–14).

There is no information about scorpion fauna of this Province and there is a big gap in the case of spatial distribution of scorpions in this area, regarding the reports of annual scorpion sting cases from North Khorasan health centers (15) and having no information about scorpions, therefore, we for the first conducted a survey to determine scorpion fauna and their spatial distribution in North Khorasan Province, northeast of Iran.

## Materials and Methods

### Study area

The North Khorasan Province has an area of 28179km^2^. It has 8 counties including Bojnurd, Shirvan, Esfarayen, Mane, Raz Jargalan, Jajarm, Faruj and Garme. The population of this province is 811,572. Geographically, this province has common borders with Turkmenistan in the north, Khorasan Razavi Province in the east and south, Semnan Province in the southwest, and Golestan Province in the west (16).

### Scorpion collection and identification

**Fig. 1.**
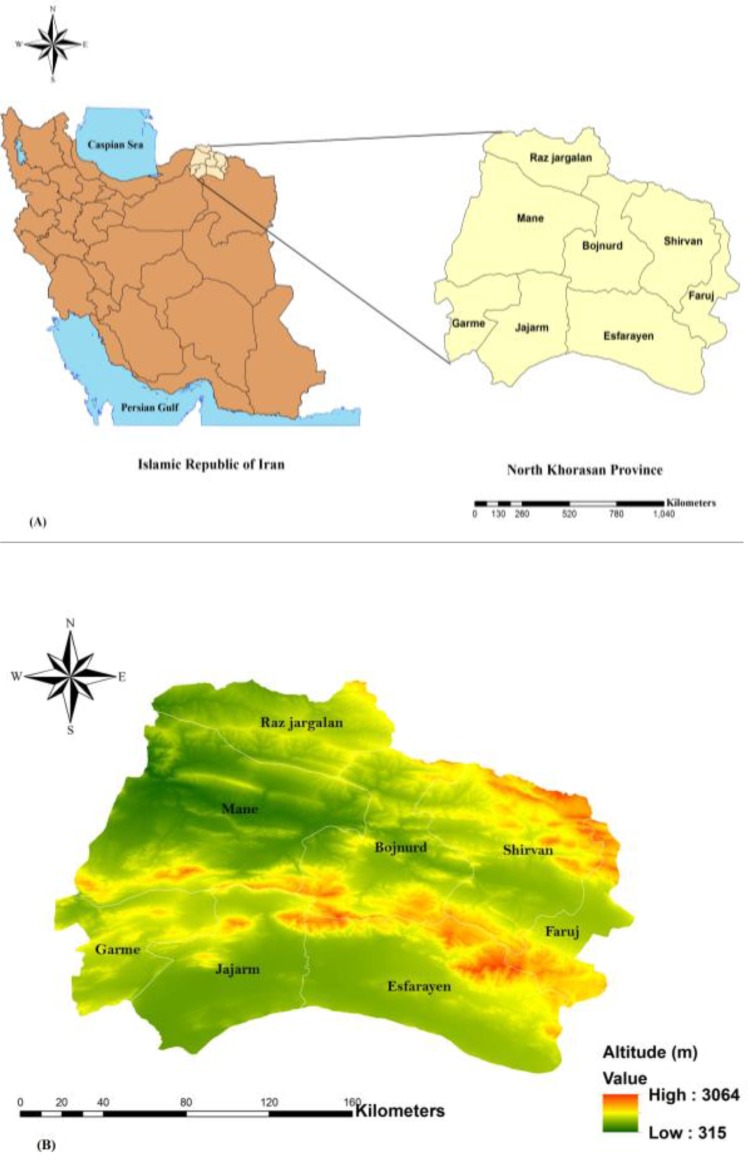
(A) The study area in North Khorasan Province, Northeast of Iran, (B) study area based on altitude

In this descriptive study, all of urban and rural areas of North Khorasan Province were investigated by considering factors such as climatic conditions (such as temperature, humidity), geographical factors (such as elevation, vegetation status, soil type, rainfall) and the different climate areas (desert, semi-desert, foothills and mountainous areas) during Jan– Dec 2017 (17). In each of the counties, two or three sites were selected as sampling locations. Locations were selected in terms of being plain or mountainous. Twenty-four locations were determined for collecting scorpions (Fig. 1). At each location, residential and non-residential areas were also taken into account. Scorpions were captured using three methods: a): ultra-violet (UV) light, in which the researchers made a search for scorpions for two hours in probable scorpions’ habitats including under the stones, under the leaves and shells of trees. At night and daytime before sunset (18). b): Pitfall traps in which plastic containers (with 8cm width and 1cm depth) were placed at stations located in the plains on a possible path of scorpion movement at a depth of 11cm of soil, and in order to draw scorpions, moist cotton was placed inside the traps (19). Ten traps were used at each location, amounting to a total of 80 traps and the scorpions were collected using iris forceps; and c): digging method by which the researchers identified probable scorpions’ habitats (underground cavities drilled by scorpions) at stations located in plain areas with soft soil and poured some water into their habitats in order to collect digger scorpions. Finally, scorpions were collected with the use of an iris forceps (20). For morphological identification, after being encoded, the collected scorpions were stored in plastic containers having 70% ethanol and then transferred to the medical entomology lab of Tehran University of Medical Sciences for species identification based on morphological keys (5).

### Statistical analysis

Arc GIS 9.3 software was used for mapping spatial distribution of scorpions.

## Results

**Fig. 2.**
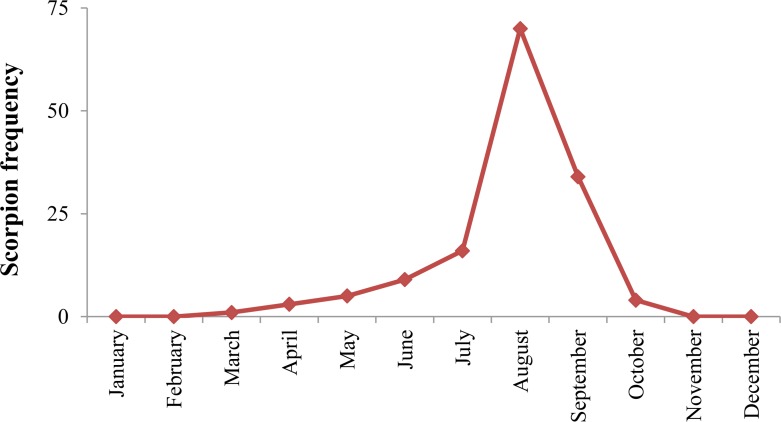
Average monthly scorpion collection, northeast of Iran, 2017

Overall, 143 scorpions were captured and identified. All collected scorpions belonged only to Buthidae family. Moreover, they were classified into four genera (*Androctonus*, *Mesobuthus*, *Odontobuthus*, *Orthochirus*) and five species namely, *M. eupeus* (59.44%), *A. crassicauda* (16.78%), *O. doriae* (12.59), *M.* (*Olivierus*) *caucasicus* (9.09%), and *O. farzanpayi* (2.10%) (Table 1). The highest frequency of scorpions (72.94%) belonged to desert areas. In addition, the majority of them (58.04%) were captured in areas with clay soil. The frequency of captured scorpion was approximately similar in urban and rural areas (48.95% in urban areas versus 51.05% in rural areas). Most of collected samples were gathered from outdoors areas (84.61%). The majority of scorpion’s samples (70 of 143) were captured in summer (August) using ultra-violet (UV) light (75.52%) method (Fig. 2).

**Table 1.** Spatial distribution of scorpions captured in northeast of Iran

**Species**															

**Location**		**Longitude** **(Decimal degree)**	**Latitude** **(Decimal degree)**	***M. eupeus***		***M. caucasicus***		***O. farzanpay***		***A. crassicauda***		***O. doriae***		**Total**	
	
**County**	**Altitude (m)**			**N**	**%**	**N**	**%**	**N**	**%**	**N**	**%**	**N**	**%**	**N**	**%**
**Garme**	2118	56.290284°	36.985493°	15	17.65	-	0.00	3	100	6	25.00	4	22.22	28	19.58
**Esfarayen**	1244	57.496673°	37.066677°	16	18.82	3	23.08	-	0.00	5	20.82	3	16.67	27	18.88
**Mane**	850	56.741207°	37.662061°	13	15.29	-	0.00	-	0.00	5	20.82	3	16.67	21	14.68
**Bojnurd**	1070	57.314313°	37.470214°	18	21.18	-	0.00	-	0.00	1	4.17	1	5.55	20	13.99
**Raz jargalan**	1279	57.109775°	37.932390°	10	11.77	-	0.00	-	0.00	4	16.68	5	27.27	19	13.29
**Faruj**	1200	58.098205°	37.085118°	5	5.88	7	53.84	-	0.00	2	8.34	1	5.56	15	10.49
**Shirvan**	1097	57.927618°	37.409236°	5	5.88	-	0.00	-	0.00	1	4.17	1	5.56	7	4.90
**Jajarm**	1000	56.371311°	36.963614°	3	3.53	3	23.08	-	0.00	-	0.00	-	0.00	6	4.19
**Total**	-	57.101294°	37.471000°	85	59.44	13	9.09	3	2.10	24	16.78	18	12.59	143	100

### 

#### Mesobuthus eupeus

**Fig. 3.**
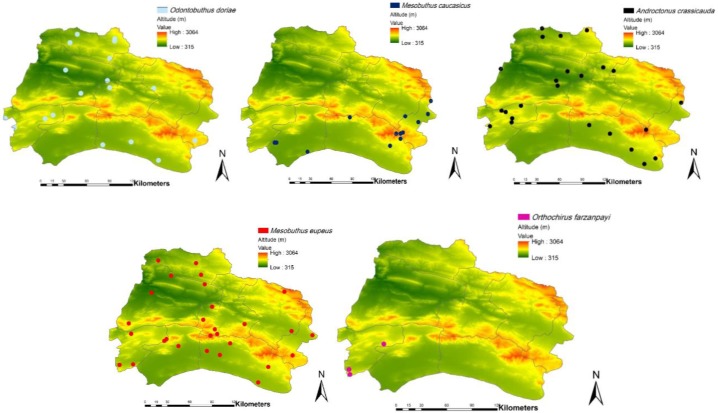
Spatial distribution of captured scorpions, northeast of Iran, 2017

Totally, 85 samples of *M. eupeus* (59.44% of the gathered specimens) were gathered*.* According to its spatial distribution, these scorpion species were collected from all 8 counties in the province; that is, from Jajarm to Raz Jargalan (Table 1, Fig. 3). It was captured in different climate areas. The other ecological characteristics of this species have been presented in Table 2. These non-digger scorpion species were collected in urban and rural regions from desert and mountainous areas which had the altitude range of 850–2118m. In this study, 75.29% of the samples were collected from outside human dwellings such as under stones and bark of trees, on the farm, around the houses, in deserts, old homes, and grasslands. The others (24.71%) were caught inside human habitats (storage place, under carpets, under furniture, inside wardrobes).

**Table 2.** Some ecological characteristic of scorpions captured in northeast of Iran

**Scorpion species Ecological characteristic**	**Different climate areas**				**Type of soil**		**Time of capture**		**Season of capture**			

	**Deserts**	**Mountains**	**Semi desert**	**Foothills**	**Sandy**	**Clay**	**Night N (%)**	**Day N (%)**	**Spring**	**Summer**	**Autumn**	**Winter**
***M. eupeus***	62 (72.94)	13 (15.30)	3 (3.53)	7 (8.23)	49 (57.65)	36 (42.35)	69 (81.18)	16 (18.82)	8	74	2	1
***A. crassicauda***	22 (91.66)	-	-	2 (8.34)	3 (12.50)	21 (87.50)	17 (70.83)	7 (29.17)	5	18	1	-
***O. doriae***	18 (100)	-	-	-	2 (11.11)	16 (88.89)	12 (66.67)	4 (33.33)	2	13	1	-
***M. caucasicus***	7 (53.84)	-	3 (23.08)	3 (23.08)	3 (13.08)	10 (76.92)	7 (53.8)	6 (46.2)	2	11	-	-
***O. farzanpayi***	3 (100)	-	-	-	-	3 (100)	3 (100)	-	-	3	-	-

#### Androctonus crassicauda

Overall, 24 samples (16.78% of the collected specimens) from all of counties were cap tured from deserts and foothills areas with the exception of Jajarm County at an altitude of 10m (Fig. 3). This species was collected both at indoor and outdoor human dwellings. This species was mostly caught in clay soil at nights and during the summer (Table 2).

#### Odontobuthus doriae

This digger scorpion species wascollected in all North Khorasan counties except Jajarm County (Fig. 3). This species was caught only outside human settlements and in plain and desert areas. The majority of this species was captured in clay soil at nights and during the summer (Table 2).

#### Mesobuthus caucasicus

This species was gathered in Jajarm, Faruj and Esfarayen Countries from desert, semi-desert and foothill areas with an altitude range of 1000 to 1244m (Fig. 3). The other ecological characteristics of this species have been presented in Table 2.

#### Orthochirus farzanpayi

This scorpion species was captured only in Garme County; the highest area of North Khorasan Province with three specimens (Fig. 3). This species was collected in desert area and in sandy soil at nights and during the summer. (Table 1, 2).

## Discussion

The results of the present investigation indicate that five scorpion species were identified in the studied areas. All of collected species belonged to Buthidae family, which is in agreement with the results of research carried out in other regions of Iran. According to studies conducted in Taibad County of Razavi Province, Darmian County of South Khorasan, Sistan, and Baluchestan (southeastern Iran), Kerman and Shiraz (southern Iran), most of captured scorpion species were of Buthidae family as well (21–23). Factors such as the high adaptability of species in this family due to different climatic conditions have led to their extensive geographical distribution in different regions, as species of this family have been widely dispersed in different areas of the world and Iran (21). Based on GIS findings, the members of this family was captured in all the counties and habitats with different climatic areas, and ecologic conditions in urban and rural areas outside human dwellings such as under stones and bark of trees, on the farm, around the houses, in deserts, old homes and grasslands.

In line with previous studies in different areas of Iran, in the current research, *M. eupeus* was the dominant species in the study area (22, 24). Additionally, in the present study *A. crassicauda* were gathered from all of counties in deserts and foothill areas except for Jajarm County. This species was collected both at indoor and outdoor human dwellings. Furthermore, this species was mostly collected in clay soil during the summer season and at nights. This species has been reported in most provinces of Iran such as Bushehr, Semnan, Khuzestan, Ilam, West Azarbaijan, Kurdistan, Khorasan Razavi, South Khorasan, Kerman, Kermanshah and Sistan and Baluchistan (25), all of which verify the results of this research. The other scorpion species collected in this present study was *M. caucasicus*. It in terms of frequency, it ranked second among the collected scorpions in North Khorasan Province. This is also in line with the results of previous studies carried out in other areas of Iran such as West Azerbaijan, Sistan and Baluchestan, Isfahan, Khorasan, Tehran, and Semnan provinces (26). The presence of this scorpion species in the current study can be confirmed by previous reports of this species in Razavi Khorasan and Sistan and Baluchestan provinces, in all of which the study areas had climatic conditions similar to that of the current study. In the present study, *O. farzanpayi* was found with very low frequency only in Garme County. This species was collected in mountainous areas and in sandy soil at nights and during summer. The size of adult *O. farzanpayi* is 3 centimeters and often distributed in desert and dry areas. Spatial distribution of this scorpion species was reported in plain and flat regions of Hormozgan and South Khorasan provinces (south and east of Iran) and has been previously proven (27). *Odontobuthus doriae* as a digger scorpion species was captured in all North Khorasan counties except in Jajarm County. This species was caught only outside human settlements and in plain and desert areas. The majority of this species was captured in clay soil at nights and during summer. This digger scorpion is able to dig a cavity up to 40 centimeters deep within the earth.

Data of the current research indicated that the scorpion activity started in March, and reached its peaked in August when the weather was favorable, leading to a gradual increase in the frequency of scorpion species. This finding was corroborated by others (21, 22). The presence of suitable environmental conditions for the emergence of species has caused the presence of scorpion species to reach its peak in the study area in August. Therefore, due to the high incidence of scorpions in the environment in Aug, the scorpion sting cases also tend to increase, which call for an urgent need to implement training programs by health-care providers in an effort to prevent scorpionism in this month. In this investigation, most of scorpions were collected at night. This in agreement with the results of research carried out in other regions of Iran such as South Khorasan Province (22, 28). According to the scientific fact that scorpions are nocturnal creatures, this finding is theoretically expected. Therefore, personal protection against scorpion stings at night be undertaken. To the current authors’ knowledge, among the limitations of this study mention can be made of the potential risks of scorpion stings and the problems of collecting scorpions in mountainous areas.

## Conclusion

We captured five scorpions’ species including *M. eupeus*, *M. caucasicus*, *O. doriae*, *A. crassicauda* and *O. farzanpayi*, among which the first four species are among most medically important scorpions in Iran that *M. eupeus* is responsible for most scorpion envenomation in this province and *A. crassicauda* is considered as most deadly one, our results showed the geographical distribution of medically important scorpions, so this information are useful to produce monovalent antivenom for scorpion sting treatment. Health education programs are implemented by health-care providers in order to prevent the occurrence of this health problem especially during the seasonal activity of scorpions in this area.

## References

[1] Dunlop JA Selden PA (2013) Scorpion fragments from the Silurian of Powys, Wales. Arachnology. 16: 27–32.

[2] Mohammadi Bavani M Rafinejad J Hanafi-Bojd AA Oshaghi MA Navidpour SH Dabiri F Badakhshan M Ghorbani E Bagheri M (2017) Spatial Distribution of Medically Important Scorpions in North West of Iran. J Arthropod Borne Dis. 11: 371–382. 29322054 PMC5758633

[3] Khaghani R Tirgari S Omrani GhA Mosavi Ivanaki A (2005) Faunistic study biodiversity of scorpions of island Kish. Modares J Med Sci. 3: 46–52.

[4] Williams SC (1987) Scorpion bionomics. Annu Rev Entomol. 32: 275–295. 3545055 10.1146/annurev.en.32.010187.001423

[5] Farzanpay RA (1990) Catalogue of the scorpions occurring in Iran, up to January 1986. Archives de l’Institut Razi, Iran Islamic Republic.

[6] Mullen G Stockwell S (2002) Scorpions (Scorpiones). Med Vet Entomol. 20: 411–423.

[7] Mashhadi I Kavousi Z Peymani P Salman Zadeh Ramhormozi Sh Keshavarz K (2017) Economic Burden of Scorpion Sting and Snake Bite from a Social Perspective in Iran, Shiraz E-Med J. 18: e57573.

[8] Dehghani R Rafinejad J Fathi B Shahi MP Jazayeri M Hashemi AA (2017) A retrospective study on Scropionism in Iran (2002–2011). J Arthropod Borne Dis. 11(2): 194–203 29062844 PMC5641608

[9] Dehghani R Charkhloo E Seyyedi-Bidgoli N Chimehi E Ghavami-Ghameshlo MA (2018) A Review on Scorpionism in Iran. J Arthropod Borne Dis. 12(4): 325–333. 30918902 PMC6423453

[10] Prendini L Wheeler WC (2005) Scorpion higher phylogeny and classification, taxonomic anarchy, and standards for peer review in online publishing. Cladistics. 21: 446–494. 10.1111/j.1096-0031.2005.00073.x 34892945

[11] Dupre G (2012) Repartition Continental des Scorpions. Arachnides, Bulletin De Terrariophilie Et De Recherches De L’A.P.C.I. (Association Pourla Connaissance des Invertébrés). 2012: 8–32.

[12] Sari A Hosseinie S (2011) History of study and checklist of the scorpion fauna (Arachnida: Scorpiones) of Iran. Progress Biol Sci. 1: 16–23.

[13] Navidpour S (2015) An annotated checklist of scorpions in south and southwestern parts of Iran. Int J Fauna Biol Stud. 2: 9–15.

[14] Dehghani R Motevali Haghi F Yousef Mogaddam M Sedaghat MM Hajati H (2016) Review study of scorpion classification in Iran. J Entomol Zool Stud. 4: 440–444.

[15] Ebrahimi V Hamdami E Moemenbellah-Fard MD Ezzatzadegan-Jahromi S (2017) Predictive determinants of scorpion stings in a tropical zone of south Iran: use of mixed seasonal autoregressive moving average model. J Venom Anim Toxins Incl Trop Dis. 23: 39. 28852405 10.1186/s40409-017-0129-4 PMC5569496

[16] Alavinia SM Arzamani K Reihani MH Jafari J (2009) Some Epidemiological Aspects of Cutaneous Leishmaniasis in Northern Khorasan Province, Iran. Iran J Arthropod Borne Dis. 3: 50–54. 22808382 PMC3385531

[17] Foord SH Gelebe V Prendini L (2015) Effects of aspect and altitude on scorpion diversity along an environmental gradient in the Soutpansberg, South Africa. J Arid Environ. 113: 114–120.

[18] Mokhayeri H Taherian S Kayedi M Navidpour S Chegeni-Sharafi A Saki M (2015) Scorpion species in trackless areas of Aligudarz and Sepiddasht Counties in Luristan Province in 2013. J Preventive Med. 1: 46–50.

[19] Nime MF Casanoves F Mattoni CI (2014) Scorpion diversity in two different habitats in the Arid Chaco, Argentina. J Insect Conservation. 18: 373–384.

[20] Vatani H Khoobdel M (2009) Scorpion fauna in Taybad region and scorpion sting status in military environment. J Mil Med. 11(1): 7–11.

[21] Motevalli Haghi F Dehghani R (2017) A Review of Scorpions Reported in Iran. J Mazandaran Univ Med Sci. 27: 213–226.

[22] Mogaddam MY Dehghani R Enayati AA Fazeli-Dinan M Vazirianzadeh B Yazdani-Cherati J Motevalli-Haghi F (2016) Scorpion Fauna (Arachnida: Scorpiones) in Darmian County, Iran (2015–2016). J Mazandaran Univ Med Sci. 26: 108–118.

[23] Nejati J Mozafari E Saghafipour A Keyhani M (2014) Scorpion fauna and epidemiological aspects of scorpionism in southeastern Iran. Asian Pac J Trop Biomed. 4: S217–S221. 25183084 10.12980/APJTB.4.2014C1323 PMC4025348

[24] Nejati J Saghafipour A Mozaffari E Keyhani A Jesri N (2017) Scorpions and scorpionism in Iran’s central desert. Acta Trop. 166: 293–298. 27923555 10.1016/j.actatropica.2016.12.003

[25] Dehghani R Moabed S Kamyabi F Haghdoost AA Mashayekhi M Soltani H (2008) Scorpions Fauna of Kerman Province, Iran. J Kerman Univ Med Sci. 2: 172–181.

[26] Mirshamsi O Sari A Hosseinie S (2011) History of study and checklist of the scorpion fauna (Arachnida: Scorpiones) of Iran. Progress in Biological Sciences. 1: 16–28.

[27] Kovarik F (1997) Results of Czech Biological Expedition to Iran part 2. Arachnida: Scorpiones, with description of Iran obuthuskrali gen. n. et sp. n and Hottentotta zagrosensis sp. n. (Buthidae). Acta Soc Zool Bohem. 61: 39–52.

[28] Ramezani Avval Riabi H Motallebi M Rafinezhad J Amiri M (2010) The ecofaunistics of scorpions in Gonabad. Horizon Med Sci. 15(4): 54–61.

